# *Clec9a-*Mediated Ablation of Conventional Dendritic Cells Suggests a Lymphoid Path to Generating Dendritic Cells *In Vivo*

**DOI:** 10.3389/fimmu.2018.00699

**Published:** 2018-04-16

**Authors:** Johanna Salvermoser, Janneke van Blijswijk, Nikos E. Papaioannou, Stephan Rambichler, Maria Pasztoi, Dalia Pakalniškytė, Neil C. Rogers, Selina J. Keppler, Tobias Straub, Caetano Reis e Sousa, Barbara U. Schraml

**Affiliations:** ^1^Walter-Brendel-Centre for Experimental Medicine, University Hospital, LMU Munich, Planegg Martinsried, Germany; ^2^Biomedical Center, LMU Munich, Planegg Martinsried, Germany; ^3^Immunobiology Laboratory, The Francis Crick Institute, London, United Kingdom; ^4^Technische Universität München, Klinikum Rechts der Isar, Institut für Klinische Chemie und Pathobiochemie, Munich, Germany; ^5^Core Facility Bioinformatics, Biomedical Center (BMC), LMU Munich, Planegg Martinsried, Germany

**Keywords:** dendritic cell, development, CLEC9A/DNGR-1, DC depletion, myelopoiesis, lymphopoiesis, fate mapping

## Abstract

Conventional dendritic cells (cDCs) are versatile activators of immune responses that develop as part of the myeloid lineage downstream of hematopoietic stem cells. We have recently shown that in mice precursors of cDCs, but not of other leukocytes, are marked by expression of DNGR-1/CLEC9A. To genetically deplete DNGR-1-expressing cDC precursors and their progeny, we crossed *Clec9a-Cre* mice to Rosa-lox-STOP-lox-diphtheria toxin (DTA) mice. These mice develop signs of age-dependent myeloproliferative disease, as has been observed in other DC-deficient mouse models. However, despite efficient depletion of cDC progenitors in these mice, cells with phenotypic characteristics of cDCs populate the spleen. These cells are functionally and transcriptionally similar to cDCs in wild type control mice but show somatic rearrangements of Ig-heavy chain genes, characteristic of lymphoid origin cells. Our studies reveal a previously unappreciated developmental heterogeneity of cDCs and suggest that the lymphoid lineage can generate cells with features of cDCs when myeloid cDC progenitors are impaired.

## Introduction

Conventional dendritic cells (cDCs) are remarkable activators of adaptive immune responses with a superior capacity to capture, process, and present antigens to T cells (1–4). Through the production of cytokines, cDCs further direct effector responses and play essential roles in immune homeostasis and innate immunity (5–10). This versatility of cDCs in immune responses is regulated in part through the functional specialization of cDC subpopulations. cDCs exist as two main developmentally distinct subsets that are found across lymphoid and non-lymphoid organs. cDC1 requires the transcription factors Batf3, IRF8, and ID2 for their development and are marked by expression of XCR1 and DNGR-1 (a.k.a. CLEC9A) across tissues and species (1, 11, 12). They are exceptional activators of CD8 T cells owing to their superior capacity to take up dead cells and cross-present antigens (1–4). Through the production of IL-12, cDC1 promotes type-I immune responses hallmarked by expression of Th1 cytokines (1, 5). In contrast, the differentiation and function of the cDC2 subset is controlled by the transcription factors IRF4, KLF4, RelB, and ZEB2 (1, 11–14), although ZEB2 may predominantly control cDC2 differentiation in inflammatory conditions (15). Phenotypically cDC2 can be distinguished from cDC1 by expression of CD11b, CD172a, and CLEC4A4 (DCIR2), as well as expression of IRF4 and concomitant lack of IRF8 (1, 11, 12, 16). cDC2 appears to play a more prominent role in CD4 T-cell activation and the promotion of Th2 and Th17 responses, particularly at mucosal surfaces (17, 18).

Although the existence of cDCs as independent cell type has been a matter of intense debate, lineage tracing and other studies in mice have established that cDCs develop as distinct hematopoietic cell lineage (12, 19, 20). Single-cell analyses have recently suggested that myeloid lineage decisions take place early in hematopoiesis but the production of lymphoid and myeloid lineages is thought to branch after the lymphoid-primed multipotent progenitor (LMPP) stage (20–24). Downstream of LMPP, in a stepwise differentiation process, common myeloid progenitors give rise to a lineage (lin) negative CD115^+^CD117^hi^CD135^+^ fraction of bone marrow that generates monocytes, plasmacytoid DCs (pDCs), and cDCs and has therefore been termed macrophage and DC progenitor (MDP) (25, 26). Whether MDPs are a true bi-potent developmental intermediate for cDCs/pDCs and monocytes is controversial (27). MDPs further give rise to common monocyte progenitors that exclusively generate monocytes (28) and a lin^−^CD115^+^CD117^low^CD135^+^ fraction of cells termed common DC progenitors (CDP) that lose monocyte potential but generate cDCs and pDCs (29, 30). Within CDPs the expression of the C-type lectin receptor DNGR-1 (encoded by the *Clec9a* gene) distinguishes cells with exclusive cDC potential (19). CDPs further differentiate into pre-cDCs, which also express DNGR-1 (19) and travel *via* blood to peripheral organs, where their terminal differentiation into cDC1 and cDC2 takes place in response to environmental cues (31–33). Some pre-cDCs exist as pre-committed subsets that can be already distinguished in bone marrow based on the expression of specific surface markers and transcription factors (32, 34, 35). The developmental steps of cDC differentiation appear conserved, as equivalent progenitors and transcriptional requirements for cDC differentiation have been identified in humans (12, 36–40).

By crossing mice expressing Cre recombinase (Cre) under the control of the endogenous *Clec9a* promoter to Rosa26-lox-STOP-lox-yellow fluorescent protein (YFP) reporter mice, we were able to demonstrate that *Clec9a* expression history faithfully traces the cDC lineage, including the main cDC1 and cDC2 subsets, but no other myeloid and lymphoid lineages in steady state as well as during inflammation (19). In this model, any cell-expressing Cre becomes irreversibly labeled with YFP, thereby allowing us to trace DNGR-1-expressing CDP and pre-cDC irrespective of continuous DNGR-1 expression (19). One exception to faithful tracing of the cDC lineage is pDCs, which do not arise from DNGR-1-expressing cDC progenitors but express low levels of DNGR-1 in their differentiated form (19). In pDCs *Clec9a* expression history, therefore, is not necessarily a measure of cell origin (19). The same applies to cDC1, which express high levels of DNGR-1 and could become labeled with YFP because they express Cre in their differentiated form (19). However, cDC1 are CDP-derived (1, 11) and arise from DNGR-1-expressing cDC progenitors upon adoptive transfer, confirming their classification as cDCs (19). Despite these limitations, *Clec9a-Cre* mice offer a powerful means to identify cDCs as descendants from *Clec9a*-expressing cDC progenitors and to trace the cDC lineage, particularly the cDC2 subtype, which lacks DNGR-1 in its differentiated form.

Although we have reached a consensus that cDCs arise as part of the myeloid lineage, several studies have suggested a lymphoid path to cDC development. Purified lymphoid progenitors can differentiate into cells resembling cDC1 and cDC2 after adoptive transfer and *in vitro* (41–47). This process appears to be driven by the same lineage promoting transcription factors that control cDC development from myeloid progenitors, such as IRF8 (46, 48). Additionally, B-cell receptor gene rearrangements at the IgH locus, indicative of a lymphoid past, can be found in populations of thymic cDCs, some splenic cDC1, and pDCs (49–52). Nevertheless, fate-mapping studies in steady-state mice have excluded a prominent contribution of lymphoid progenitors to the steady-state cDC pool (53, 54) and have confirmed a binary branching of lymphoid and myeloid lineages downstream of hematopoietic stem cells (21). However, whether lymphoid progenitors can serve as an alternative path to DC poiesis in conditions of inflammation or when myeloid cDC progenitors are absent has not been investigated. Because cDCs generated *in vitro* from purified human lymphoid or myeloid progenitors are indistinguishable by gene expression analysis (42), addressing this question requires faithful ontogeny-based fate-mapping models.

Here, we investigated cDC development in mice in conditions in which cDC progenitors are impaired. We crossed *Clec9a-Cre* mice to Rosa26-lox-STOP-lox-diphtheria toxin (DTA) reporter mice (55) (*Clec9a^Cre^Rosa^DTA^*) to constitutively deplete *Clec9a*-expressing cDC progenitors and their progeny. We found that *Clec9a^Cre^Rosa^DTA^* mice lack cDC progenitors and cDC1 but not cDC2. We show that in the absence of cDC progenitors, cells with features of cDC2 arise *via* an alternative developmental path. These cells show similarities to bona fide cDC2 in terms of transcriptional profile and inflammatory cytokine production but exhibit evidence of Ig receptor rearrangements, indicating a lymphoid origin. Thus, our data suggest a previously unrecognized role for lymphoid progenitors as an alternative source of cDC2, when the conventional myeloid path of cDC development is blocked.

## Materials and Methods

### Mice

*Clec9a-Cre* (19), Rosa26-lox-STOP-lox-EYFP (56), Rosa26-lox-STOP-lox-DTA (55), Rosa26-lox-STOP-lox-DTR (57), C57BL/6J, and B6.SJL mice were bred at Cancer Research UK, at ENVIGO or the Biomedical Center in specific pathogen-free conditions. All animal experiments were performed in accordance with national and institutional guidelines for animal care and approved by the Francis Crick Institute Animal Ethics Committee, the UK Home Office, or the Regierung of Oberbayern.

### Cell Isolation

Spleen and lymph nodes were cut into small pieces and digested with Collagenase IV (200 U/mL; Worthington) and DNase I (0.2 mg/mL Roche) in RPMI for 30 min at 37°C. Cells were strained through 70-µm cell strainers (BD Biosciences), washed with FACS buffer [PBS, 1% fetal calf serum (FCS), 2.5-mM EDTA, 0.02% sodium azide] and incubated for 2 min in red blood cell lysis buffer (Sigma). Cells were then washed and resuspended in FACS buffer. Bone marrow was isolated by flushing one femur with FACS buffer onto a cell strainer. Erythrocytes were lysed as above. Colonic single-cell suspensions were prepared as published (19).

### Cell Culture

CD11c^+^ cells from spleen were enriched by positive selection using magnetic beads and LS-columns (Miltenyi), cultured in complete RPMI (10% FCS, penicillin/streptomycin, non-essential amino acids, sodium pyruvate, L-glutamine, 0.025-mM β-mercaptoethanol), and stimulated with LPS (100 ng/mL) or CpG (0.5 µg/mL) for 2 h before addition of brefeldin A (5 µg/mL) for an additional 4 h.

### ELISA

FLT3L and G-CSF were measured with DuoSet mFLT3L (DY427) and Quantikine^®^ ELISA kits (both R&D Systems) according to the manufacturer’s recommendations. Other cytokines were measured by Legendplex (BioLegend).

### Flow Cytometry

Data were collected on a LSR Fortessa (BD Biosciences) and analyzed with FlowJo software (Tree Star, Inc.). Cell sorting was performed on an Aria III Fusion (BD Biosciences). Antibodies used in this study can be found in Table S2 in Supplementary Material. For intracellular cytokine staining cells were fixed with 2% paraformaldehyde (15 min, room temperature), then washed in FACS buffer with 0.05% saponin and stained in 0.5% saponin. IRF8 and IRF4 were stained in Foxp3 transcription factor staining set (eBioscience-00-5523-00) and Zbtb46 in transcription factor buffer from BDBioscience-562574. Dead cells were identified with Dapi or live/dead fixable violet (Invitrogen) or zombie UV dye (Biolegend).

### Microarray Analysis

CD11c^+^ cells were enriched using magnetic beads and LS columns (Miltenyi Biotec). Cells were then sorted as CD11c^+^MHCII^+^CD11b^+^CD24^−^CD64^−^Gr-1^−^ cells from *Clec9a^cre/cre^Rosa^DTA^* mice or as YFP^+^CD11c^+^MHCII^+^CD11b^+^CD24^−^CD64^−^Gr-1^−^ cells from *Clec9a^cre/cre^Rosa^YFP^* mice. Total RNA was isolated using Qiagen RNeasy Micro Kit and prepared for hybridization on Affymetrix Mouse Gene 1.0 ST Arrays. We processed microarray data with R/bioconductor (R version 3.4.2) using the annotation package “mogene10sttranscriptcluster.db” version 8.7.0. The Robust Multichip Average (RMA) algorithm was used to extract raw expression values (library “oligo,” version 1.42.0 and “pd.mogene.1.0.st.v1” version 3.14.1). We subsequently removed probe sets with zero variance and kept the ones with a median log2 expression level higher than 4.5 in at least one condition. Many-to-one probe-sets-to-gene relationships were resolved by retaining per gene only the probe set with the highest interquartile range of expression levels across the samples. External data were merged and batch corrected using ComBat (library “sva,” version 3.26.0). Principle component analysis (PCA) was performed on 50% of the genes defined by highest variance across all samples. Heatmaps were generated using function pheatmap (library pheatmap, version 1.0.8) including gene-based scaling and clustering.

### DJ-Rearrangement Polymerase Chain Reaction (PCR)

Genomic DNA was extracted by phenol chloroform extraction and 20-ng DNA were used per reaction. PCR for the IgH locus (Dfl16 and Dsp2 D gene families) (58) was split in two reactions for Germline (J3 & Mu0) and DJ-rearrangement (DH L & J3).

DHL-GGAATTCG(AorC)TTTTTGT(CorG)AAGGGATCTACTACTGTG;
Mu0-CCGCATGCCAAGGCTAGCCTGAAAGATTACC; J3-GTCTAGATTCTCACAAGAGTCCGATAGACCCTGG. IgH DJ-rearrangements for the D_H_Q52 element were assessed by sequential PCRs with the following primers:
PCR-1: DHQ52-1-CACAGAGAATTCTCCATAGTTGATAGCTCAG;DHQ52-2GCCTCAGAATTCCTGTGGTCTCTGACTGGT; PCR-2: JH4-1-AGGCTCTGAGATCCCTAGACAG; JH4-2- GGGTCTAGACTCTCAGCCGGCTCCCTCAGGG as previously described (51).

### Diphtheria Toxin (DT)-Mediated Cell Ablation

Mice were injected intraperitoneally with 25 ng per gram body weight DT (SIGMA). Spleens were analyzed 24 h later.

### Statistical Analysis

Statistical testing was performed using two-sided, unpaired Welch *t*-tests in GraphPad Prism 6 software (GraphPad, La Jolla, CA, USA). A *p* < 0.05 was considered significant.

## Results

### *Clec9a^Cre/Cre^Rosa^DTA^* Mice Develop Myeloproliferative Disease

**Figure 1:**
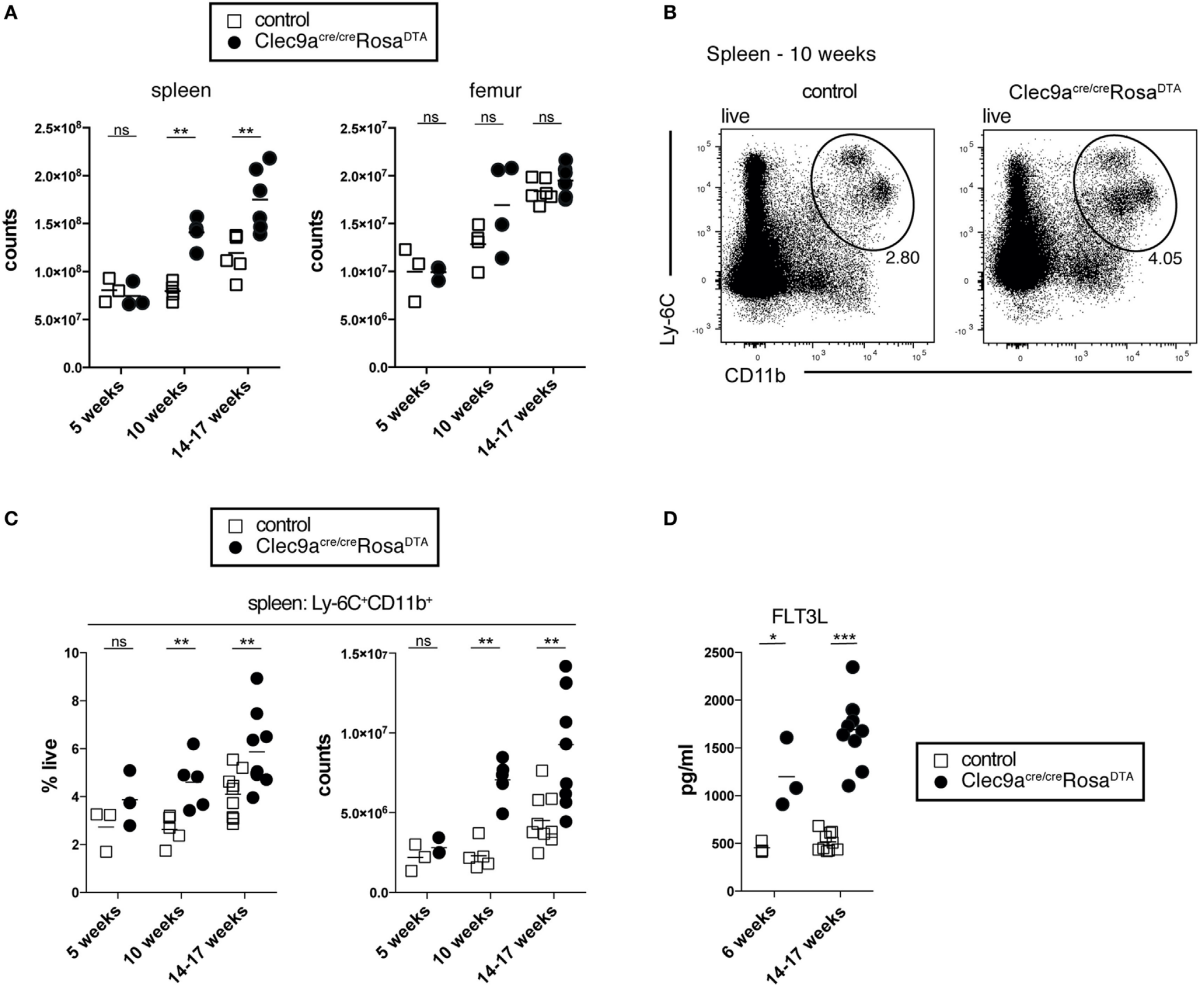
*Clec9a^cre/cre^Rosa^DTA^* mice showing signs of age-dependent myeloproliferation. **(A)** The total number of leukocytes per spleen and femur from control and *Clec9a^cre/cre^Rosa^DTA^* mice of the indicated ages is shown. **(B,C)** Ly-6C^+^CD11b^+^ cells were identified by flow cytometry in spleen from control and *Clec9a^cre/cre^Rosa^DTA^* mice. **(B)** Representative gating strategy of Ly-6C^+^CD11b^+^ cells in 10-week-old mice. Plots are gated on DAPI^−^ live cells. **(C)** Frequency and total counts of Ly-6C^+^CD11b^+^ cells in mice of the indicated ages and genotypes. **(D)** Serum was collected from control and *Clec9a^cre/cre^Rosa^DTA^* mice at 6 and 14–17 weeks of age and the concentrations of FLT3L were determined by ELISA. Each symbol represents one mouse. ns, not significant; * *p* < 0.05, ** *p* < 0.001, and *** *p* < 0.0001.

To generate mice lacking progeny derived from cDC-restricted progenitors, we crossed *Clec9a-Cre* mice to Rosa26-lox-STOP-lox-DTA reporter mice to induce apoptotic cell death in Cre-expressing cells and their progeny (55). We have previously shown that the penetrance of Cre-mediated recombination is increased in homozygous *Clec9a-Cre* mice without affecting specificity (19). For most experiments, we therefore generated mice homozygous for the *Clec9a-Cre* locus that were either heterozygous or homozygous for the Rosa26-lox-STOP-lox-DTA allele (henceforth referred to as *Clec9a^Cre/Cre^Rosa^DTA^* mice). *Clec9a^Cre/Cre^Rosa^DTA^* mice were born at Mendelian ratio and developed normally (not shown). With age, spleens from *Clec9a^Cre/Cre^Rosa^DTA^* mice appeared larger (not shown) and exhibited significantly increased cellularity compared to spleens from littermate controls (*Clec9a^+/+^Rosa^DTA^* or *Clec9a^Cre^Rosa^+/+^* mice, henceforth referred to as controls, Figure 1A). Despite prominent splenomegaly in mice 10 weeks or older, no difference in bone-marrow cellularity was observed (Figure 1A). DC deficiency is associated with myeloproliferation leading to systemic neutrophilia and monocytosis (59–62). In 5-week-old mice, we observed no differences in the frequency and number of Ly-6C^+^CD11b^+^ myeloid cells but in mice 10 weeks or older Ly-6C^+^CD11b^+^ cells were significantly increased (Figures 1B,C). This age-dependent increase in Ly-6C^+^CD11b^+^ cells encompassed both neutrophils and monocytes, whereas red pulp macrophages were not affected (Figures S1A–C in Supplementary Material). Myeloproliferation in DC-deficient mice is thought to be secondary to dysregulation of growth factors, such as FLT3L, G-CSF, or GM-CSF (59, 61). *Clec9a^Cre/Cre^Rosa^DTA^* mice displayed increased serum levels of FLT3L, with a slight increase evident even before the onset of overt splenomegaly in 6-week-old mice (Figure 1D). Systemic levels of other cytokines implicated in neutrophil and monocyte homeostasis were unaffected (Figure S1D in Supplementary Material). Of note, we could not detect GM-CSF in serum of control or *Clec9a^Cre/Cre^Rosa^DTA^* mice (unpublished observation). Therefore, *Clec9a^Cre/Cre^Rosa^DTA^* mice develop splenomegaly and exhibit increased levels of FLT3L, reminiscent of the myeloproliferative disease reported in other cDC-deficient animal models (59, 61–63).

### Loss of cDC1 but Not cDC2 in *Clec9a^Cre/Cre^Rosa^DTA^* Mice

**Figure 2:**
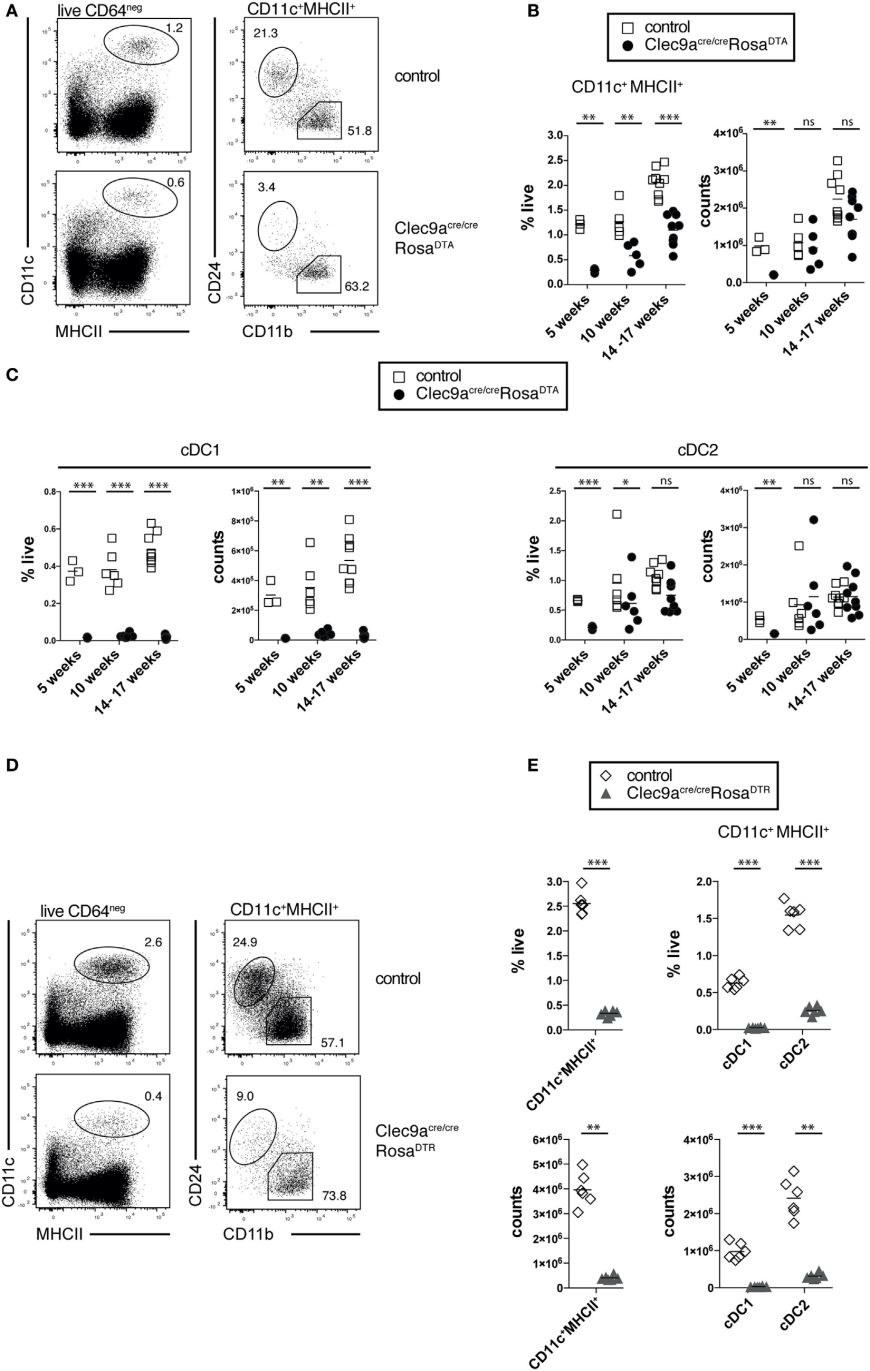
Loss of cDC1 but not cDC2 in spleen from *Clec9a^cre/cre^Rosa^DTA^* mice. **(A–C)** CD11c^+^MHCII^+^ conventional dendritic cells (cDCs) were identified by flow cytometry in spleen from *Clec9a^cre/cre^Rosa^DTA^* and control mice and further analyzed for CD24 and CD11b expression, to identify cDC1 and cDC2, respectively. **(A)** Representative gating strategy to identify CD11c^+^MHCII^+^ cDCs, CD24^+^ cDC1, and CD11b^+^ cDC2. **(B)** Frequency and total counts of CD11c^+^MHCII^+^ cells in spleen from control and *Clec9a^cre/cre^Rosa^DTA^* mice of the indicated ages. **(C)** Frequency and total counts of CD24^+^ cDC1 and CD11b^+^ cDC2 in mice of the indicated ages. **(D,E)** *Clec9a^cre/cre^Rosa^DTR^* and control mice were injected i.p. with DT and 24 h later spleens were analyzed by flow cytometry to identify CD11c^+^MHCII^+^ cells, as well CD24^+^ cDC1 and CD11b^+^ cDC2 **(D)**. **(E)** The frequency and counts of total cDCs and cDC subsets identified as in **(D)**. Each symbol represents one mouse. ns, not significant; * *p* < 0.05, ** *p* < 0.001, and *** *p* < 0.0001.

To analyze the efficiency of cDC depletion in *Clec9a^Cre/Cre^Rosa^DTA^* mice, we focused on steady-state spleen, where cDCs can reliably be identified as CD11c^+^MHCII^+^ cells and the two main cDC1 and cDC2 subsets can be distinguished as CD24^+^ and CD11b^+^ cells, respectively (Figure 2A) (12). At 10 weeks of age *Clec9a^Cre/Cre^Rosa^DTA^* mice showed a twofold reduction in the frequency of splenic cDCs compared to controls. Due to the increased organ cellularity, however, there was no effect on the absolute number of splenic CD11c^+^MHCII^+^ cells (Figures 2A,B). The same was observed in older *Clec9a^Cre/Cre^Rosa^DTA^* mice (14–17 weeks; Figure 2B). In 5-week-old mice that do not exhibit increased spleen cellularity, a reduction of splenic CD11c^+^MHCII^+^ cells was apparent in cell counts (Figure 2B), yet cell depletion was surprisingly inefficient (about 3.5-fold). When we further separated CD11c^+^MHCII^+^ cells into cDC1 and cDC2 using the surface markers CD24 and CD11b (Figure 2A), we found that cDC1 were completely depleted in spleens from *Clec9a^Cre/Cre^Rosa^DTA^* mice at all ages examined (Figure 2C and unpublished observations). In contrast, CD11b^+^ cDC2 showed a significant but small reduction (Figure 2C) only in 5-week-old *Clec9a^Cre/Cre^Rosa^DTA^* mice and their frequency and absolute number was not reduced in mice 10 weeks or older (Figure 2C). This could be seen in other lymphoid organs, as well as in mice heterozygous for Cre expression, as the lymph nodes of *Clec9a^+/Cre^Rosa^DTA^* mice also lacked cDC1 but not resident cDC2 or migratory CD103^+^CD11b^+^ and CD103^-^ CD11b^+^ cDC2 (Figure S2F in Supplementary Material). Finally, these findings could be extended to non-lymphoid organs as cDC1 but not CD103^+^CD11b^+^ or CD103^−^ CD11b^+^ cDC2 were absent from colon of *Clec9a^Cre/Cre^Rosa^DTA^* mice (Figure S2G in Supplementary Material).

The lack of cDC2 depletion was surprising, as this population shows near complete fluorescent reporter labeling in Clec9a*^Cre/Cre^*Rosa^YFP^ mice (19) and can be depleted with DT in *Clec9a-Cre* mice crossed to Rosa26-lox-STOP-lox-diphtheria toxin receptor mice (DTR; *Clec9a^Cre/Cre^Rosa^DTR^*) (64). We confirmed that a single injection of DT was sufficient to efficiently deplete pre-cDCs, cDC1, and cDC2 in *Clec9a^Cre/Cre^Rosa^DTR^* mice (Figures 2D,E; Figures S2A,B in Supplementary Material). Of note, 24 h after DT injection no increase in splenic neutrophils or monocytes was observed (Figure S2C in Supplementary Material), suggesting that *Clec9a^Cre/Cre^Rosa^DTR^* mice do not develop acute monocytosis and neutrophilia (62). Therefore, *Clec9a-Cre* mice serve as an efficient model to transiently deplete cDC1 and cDC2 when crossed to Rosa^DTR^ mice (64) but depletion of cDC2 is not seen when crossed to Rosa^DTA^ mice.

Fully differentiated pDCs express low levels of DNGR-1 although they do not arise from DNGR-1-expressing CDPs (19). Splenic pDC numbers were not altered in *Clec9a^Cre/Cre^Rosa^DTA^* mice (Figure S2D in Supplementary Material). However, pDCs that developed in *Clec9a^+/Cre^Rosa^DTA^* mice almost completely lacked DNGR-1 (Figure S2E in Supplementary Material). These data suggest that DNGR-1-expressing pDCs are depleted and replaced with DNGR-1 negative pDCs. We conclude that constitutive depletion of DNGR-1-expressing cells in *Clec9a^Cre/Cre^Rosa^DTA^* adult mice leads to a complete loss of cDC1 but not cDC2 or pDCs.

### Efficient Depletion of Pre-cDCs in Bone Marrow and Spleen of *Clec9a^Cre/Cre^Rosa^DTA^* Mice

**Figure 3:**
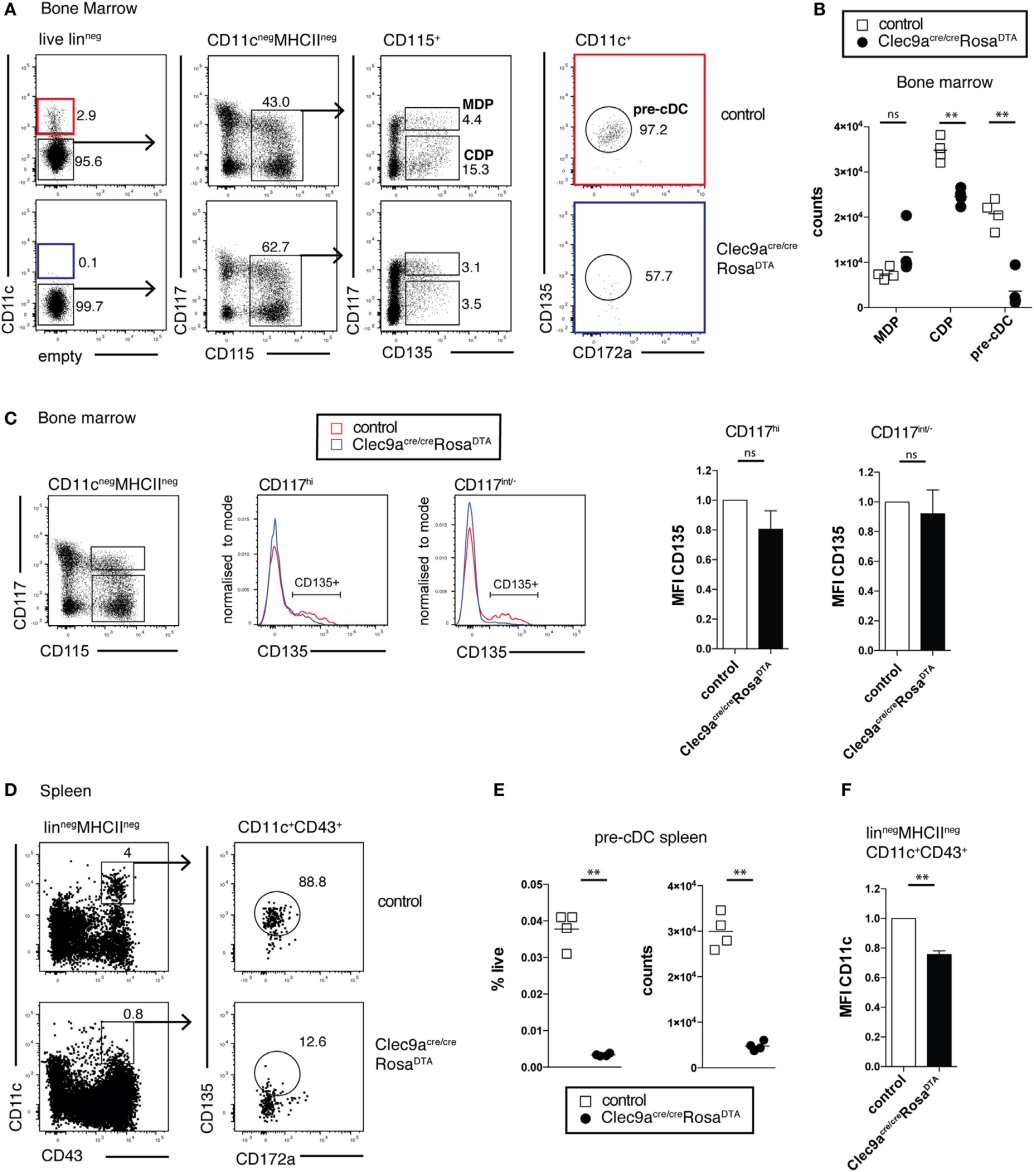
Efficient depletion of conventional dendritic cell (cDC) progenitors in bone marrow and spleen. **(A,B)** cDC progenitors were identified by flow cytometry in the bone marrow of 10-week-old control and *Clec9a^cre/cre^Rosa^DTA^* mice. **(A)** Live lineage negative (lin^−^; CD3, CD4, CD8, CD11b, MHCII, Ter119, NK1.1, and B220) cells were gated and further analyzed for CD11c, CD117, CD115, CD172a, and CD135 expression. Macrophage and DC progenitor (MDPs) were identified as CD11c^−^CD115^+^CD135^+^CD117^hi^ cells, common DC progenitors (CDPs) as CD11c^−^CD115^+^CD135^+^CD117^int/−^ cells, and pre-cDCs as CD11c^+^CD172a^int^CD135^+^ cells. **(B)** The number of MDP, CDP, and pre-cDC per femur is shown. **(C)** Lin^−^CD115^+^ CD117^hi^ and lin^−^CD115^+^CD117^int/−^ bone-marrow cells from control and *Clec9a^cre/cre^Rosa^DTA^* mice were gated and further analyzed for CD135 expression. Mean fluorescence intensity (MFI) (*n* = 4) of the CD135^+^ cell fraction, as gated in the histogram overlays, is shown. It was normalized to MFI on the same population from control mice, which is set as 1. **(D–F)** Pre-cDCs in spleen from control and *Clec9a^cre/cre^Rosa^DTA^* mice 10 weeks of age were identified as lin^−^CD11c^+^CD43^+^CD172a^int^CD135^+^ cells by flow cytometry **(D)**. **(E)** The frequency and number of pre-cDCs per spleen is shown. **(F)** CD11c MFI on splenic lin^−^MCHII^−^CD11c^+^CD43^+^ cells from *Clec9a^cre/cre^Rosa^DTA^* mice is shown. It was normalized to MFI on the same population from control mice, which is set as 1 (*n* = 4). Each symbol represents one mouse. ns, not significant; * *p* < 0.05, ** *p* < 0.001, and *** *p* < 0.0001.

We next investigated whether cDC progenitors were depleted in *Clec9a^Cre/Cre^Rosa^DTA^* mice. As expected, *Clec9a^Cre/Cre^Rosa^DTA^* mice showed no reduction in MDPs lin^−^ CD11c^−^CD115^+^CD135^+^CD117^hi^ compared to control mice (Figures 3A,B). In contrast, CDPs (lin^−^CD11c^−^CD115^+^CD135^+^CD117^low/−^) were significantly reduced in *Clec9a^Cre/Cre^Rosa^DTA^* mice (Figures 3A,B). High serum levels of FLT3L in *Clec9a^Cre/Cre^Rosa^DTA^* mice could have led to downregulation of CD135 in MDPs or CDPs, causing them to be missed in our gating. This was not the case because bone marrow lin^−^CD11c^−^CD115^+^CD117^hi^ and lin^−^CD11c^−^CD115^+^CD117^low/−^ cells from control and *Clec9a^Cre/Cre^Rosa^DTA^* mice expressed similar levels of CD135 (Figure 3C). Pre-cDCs, identified as lin^−^CD11c^+^CD135^+^CD172a^low^ cells, were also nearly absent in bone marrow of *Clec9a^Cre/Cre^Rosa^DTA^* mice when compared to controls (Figures 3A,B) and this was true when pre-cDC were also identified irrespective of the CD135 marker as the lin^−^CD11c^+^ fraction of bone marrow (Figure S3A in Supplementary Material). Similarly, in spleen, lin^−^CD11c^+^CD43^+^CD135^+^CD172a^low^ pre-cDCs were virtually absent in *Clec9a^Cre/Cre^Rosa^DTA^* mice, as well as heterozygous *Clec9a^+/Cre^Rosa^DTA^* mice (Figures 3D,E; Figure S3C in Supplementary Material). Although CD43^+^CD135^−^ cells were found in the splenic pre-cDC gate, those cells expressed reduced levels of CD11c, indicating that they were contaminants rather than bona fide pre-cDCs (Figures 3D–F; Figures S3B,C in Supplementary Material). A more efficient depletion of pre-cDCs compared with CDPs is to be expected because Cre-mediated DNA rearrangement in pre-cDCs is higher than in CDPs, reflecting the developmental hierarchy of DNGR-1 expression in rapidly cycling progenitors (19). Thus, our data indicate efficient depletion of cDC progenitors in bone marrow and spleen of *Clec9a^Cre/Cre^Rosa^DTA^* mice and suggest that cells resembling cDC2 in *Clec9a^Cre/Cre^Rosa^DTA^* mice arise independently of the classical CDP and pre-cDC differentiation pathway.

### cDC2 From *Clec9a^Cre/Cre^Rosa^DTA^* Mice Phenotypically and Functionally Resemble cDCs

**Figure 4:**
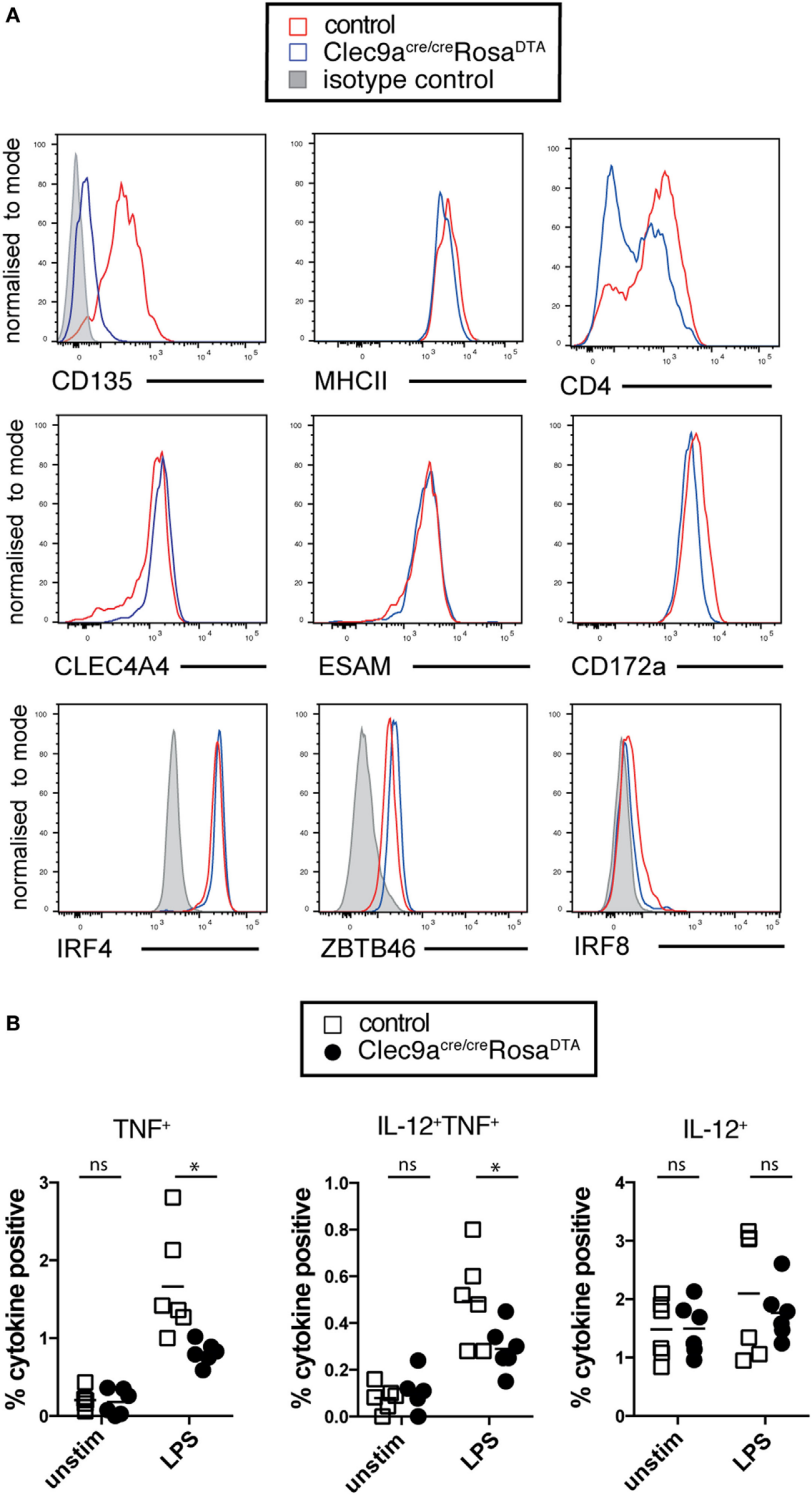

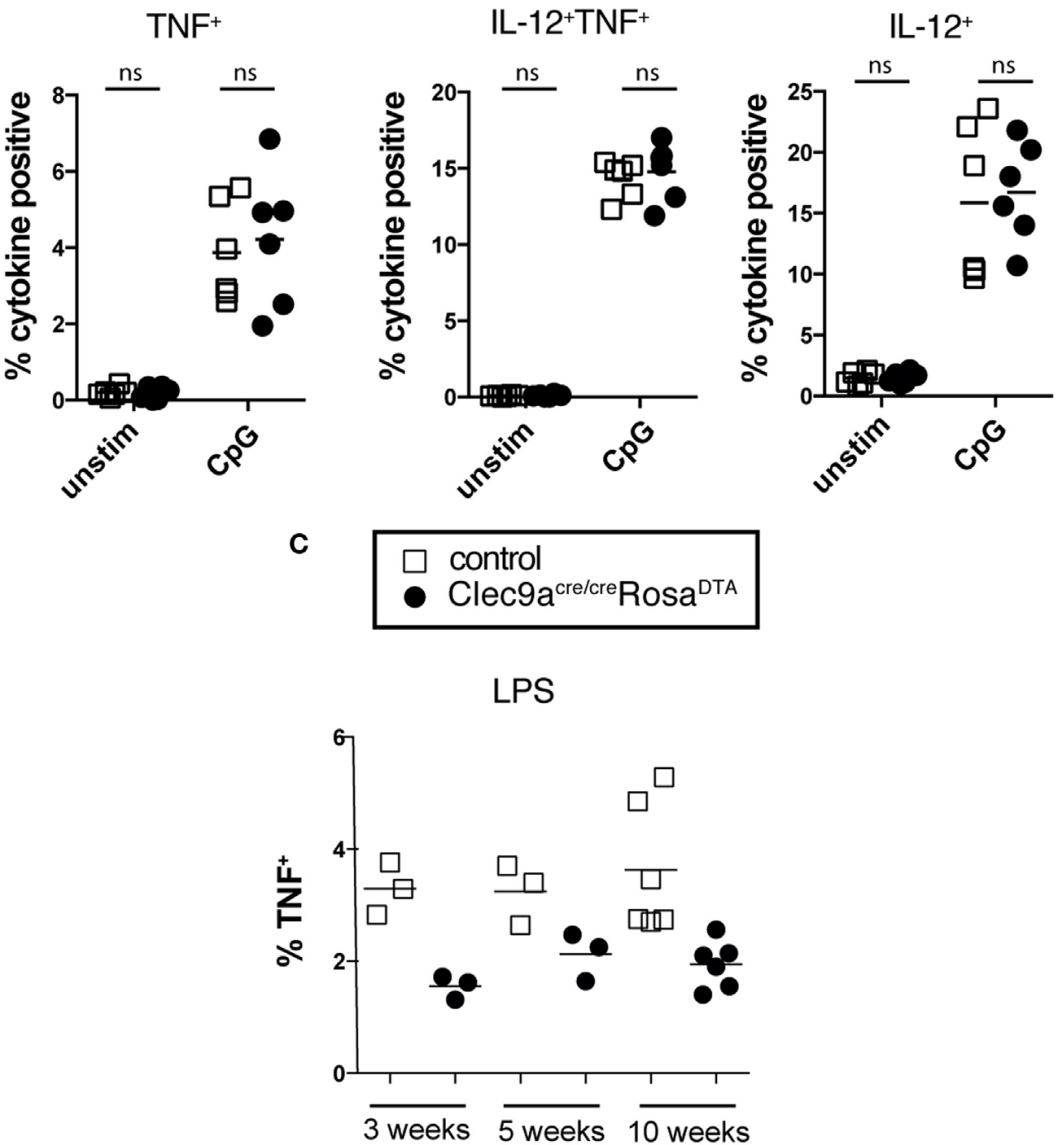
cDC2 from *Clec9a^Cre/Cre^Rosa^DTA^* mice phenotypically and functionally resemble cDCs. **(A)** Splenic CD11c^+^MHCII^+^CD11b^+^ cells from control (red) and *Clec9a^cre/cre^Rosa^DTA^* (blue) mice were analyzed for the expression of indicated surface markers or transcription factors (lower line) by flow cytometry. Gray traces represent staining with isotype matched control antibody. **(B,C)** CD11c^+^ cells from control and *Clec9a^cre/cre^Rosa^DTA^* mice of the indicated ages were enriched using magnetic beads, stimulated with lipopolysaccharide (LPS) (100 ng/mL) or CpG (0.5 µg/mL) for 6 h followed by intracellular cytokine staining. CD11c^+^MHCII^+^CD11b^+^ cDC2 were identified by flow cytometry and TNF and IL-12 production was analyzed. **(B)** The frequency of cytokine positive cDC2 is shown. **(C)** Frequency of TNF producing cDC2 from mice of the indicated ages stimulated with LPS. Each data point represents one mouse. Data are compiled from two independent experiments. * *p* < 0.05.

Phenotypic analysis revealed that cDC2 from *Clec9a^Cre/Cre^Rosa^DTA^* mice showed no alterations in surface expression of the cDC markers CD172a, MHCII, CLEC4A4, and ESAM, although the expression of CD4 was slightly lower on cDC2 from *Clec9a^Cre/Cre^Rosa^DTA^* mice compared to controls (Figure 4A). cDC2 from *Clec9a^Cre/Cre^Rosa^DTA^* mice further showed normal expression of lineage defining transcription factors ZBTB46, IRF4 and IRF8 (Figure 4A). cDC2 from control and *Clec9a^Cre/Cre^Rosa^DTA^* mice responded similarly to stimulation with the TLR ligands lipopolysaccharide (LPS) and CpG oligonucleotides (Figure 4B). Interestingly, upon LPS treatment fewer cDC2 from *Clec9a^Cre/Cre^Rosa^DTA^* mice than from control mice produced TNF, whereas this was not the case for IL-12 (Figure 4B). Reduced TNF production after LPS stimulation was already present in cDC2 from young *Clec9a^Cre/Cre^Rosa^DTA^* mice that did not yet display myeloproliferation (Figure 4C) indicating that it was not merely a functional adaptation to the myeloproliferative environment. We conclude that cDC2 from *Clec9a^Cre/Cre^Rosa^DTA^* mice are broadly similar to cDC2 from control mice, other than a slight impairment in the production of TNF after LPS stimulation.

### cDC2 From *Clec9a^Cre/Cre^Rosa^DTA^* Mice Are Transcriptionally Similar to Bona Fide cDC2 but Exhibit Somatic Rearrangements of Lymphoid Receptor Genes

**Figure 5:**
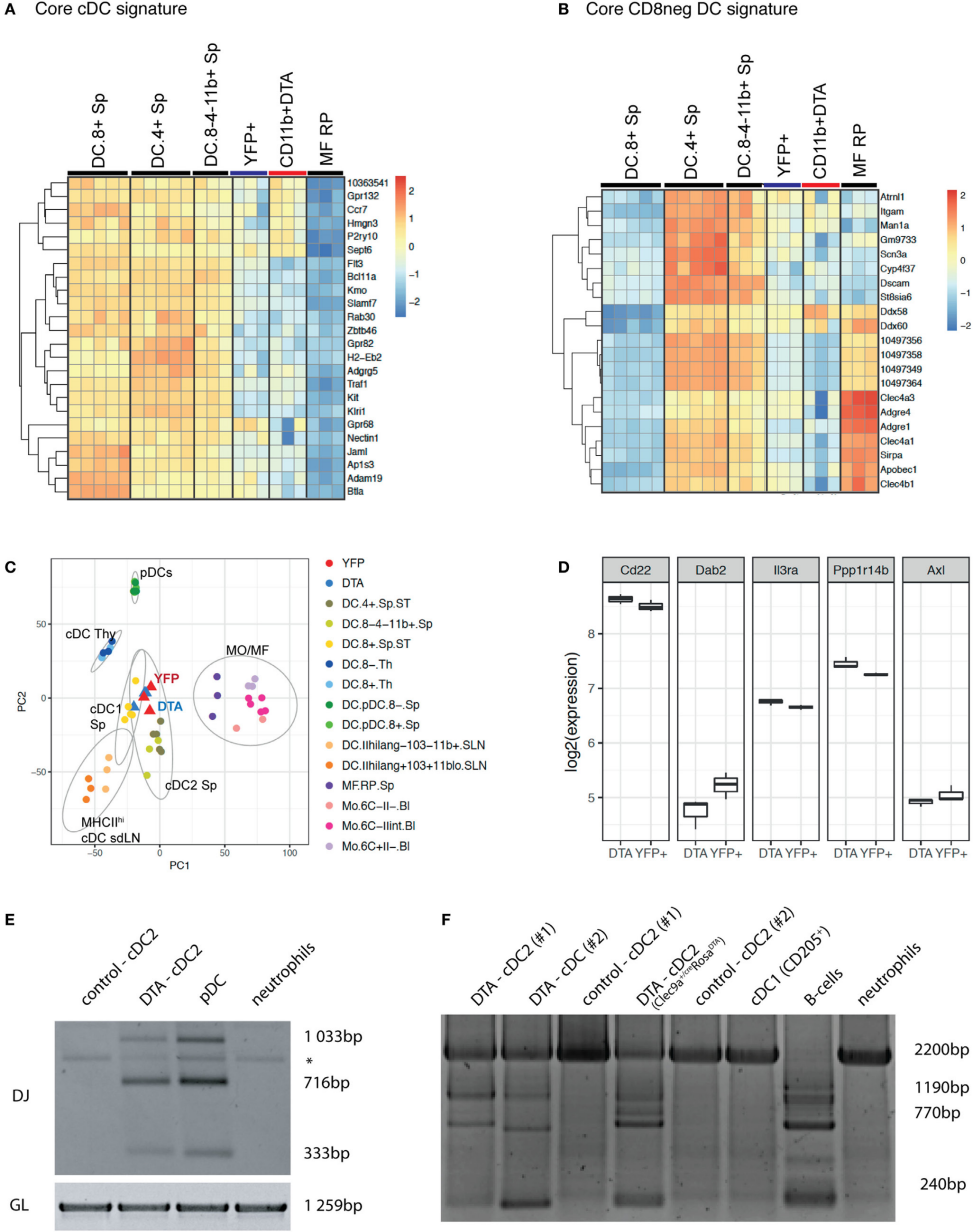
cDC2 from *Clec9a^Cre/Cre^Rosa^DTA^* mice are transcriptionally similar to bona fide cDC2 but exhibit somatic rearrangements of lymphoid receptor genes. Splenic CD11c^+^MHCII^+^CD11b^+^ cDC2 from *Clec9a^cre/cre^Rosa^DTA^* mice (CD11b^+^ DTA) and CD11c^+^MHCII^+^CD11b^+^YFP^+^ cDC2 (YFP^+^) from *Clec9a^cre/cre^Rosa*^YFP^ mice were sorted and subjected to microarray analysis. **(A,B)** Population clustering and heat map display of the relative expression values for core conventional dendritic cells (cDC) signature genes **(A)** and core CD8^neg^ cDC signature genes **(B)** comparing cDC2 from *Clec9a^cre/cre^Rosa^DTA^* mice and YFP^+^ cDC2 to the indicated murine cDC and macrophage populations from the Immgen database. **(C)** Principle component analysis (PCA) was performed on 50% of the genes defined by highest variance across all samples. Each dot of the same color represents a replicate sample. For each cluster, normal confidence ellipses are indicated. **(D)** Box plots of the log_2_ expression values of the indicated genes in cDC2 from *Clec9a^cre/cre^Rosa^DTA^* mice and YFP^+^ cDC2. **(E,F)** CD11c^+^MHCII^+^CD11b^+^ cDC2 from control and *Clec9a^cre/cre^Rosa^DTA^* mice were sorted and genomic DNA was isolated. **(E)** Polymerase chain reaction (PCR) was performed using primers for the germline (GL) locus and primer mixtures homologous for regions of the Dfl16 and Dsp2 D gene families for detecting D–J rearrangements of the IgH chain. DJ rearrangements in pDCs (SiglecH^+^B220^+^) and neutrophils (Ly-6G^+^) are shown as control. * indicates an unspecific band. **(F)** Genomic PCR was performed using primers for the GL locus and primer sets for the D_H_Q52 element for detecting D–J rearrangements of the IgH chain. DJ rearrangements in pDCs (SiglecH^+^B220^+^), CD205^+^ cDC1, and neutrophils (Ly-6G^+^) are shown as control. Each lane in E and F represents independent replicates from different mice.

We next performed whole transcriptome profiling by microarray of cDC2 from *Clec9a^Cre/Cre^Rosa^DTA^* mice. To this end, we sorted splenic CD11c^+^MHCII^+^CD11b^+^ cDC2 from *Clec9a^Cre/Cre^Rosa^DTA^* mice (CD11b^+^ *DTA*) and compared their mRNA profile to that of cDC2 sorted as YFP^+^CD11c^+^MHCII^+^CD11b^+^ cells from *Clec9a^Cre/Cre^Rosa^YFP^* mice (YFP^+^; Figure 5; Figure S3 and Table S1 in Supplementary Material). We chose this experimental strategy to ensure comparison to bona fide cDC2 arising from *Clec9a*-expressing myeloid cDC progenitors. Our analysis revealed surprisingly few differences in gene expression, indicating that cDC2 from *Clec9a^Cre/Cre^Rosa^DTA^* mice are very similar to YFP^+^ cDC2. Interestingly, among the 50 most significantly differentially expressed genes, only 11 showed more than a twofold difference and we found CD135 as a top hit (Table S1 in Supplementary Material). The expression of CD135 was about twofold lower in cDC2 from *Clec9a^Cre/Cre^Rosa^DTA^* mice than in YFP^+^ cDC2 (Table S1 in Supplementary Material), raising the possibility that expression of the lineage defining marker CD135 may be dysregulated at the mRNA level. Consistent with that notion, staining for CD135 was reduced on cDC2 from *Clec9a^Cre/Cre^Rosa^DTA^* mice (Figure 4A). However, incubation of cDCs with FLT3L inhibited CD135 staining intensity, even in conditions in which endocytosis should be blocked (Figure S3D in Supplementary Material), suggesting that, when occupied by FLT3L, CD135 may no longer be freely available for antibody binding. Therefore, decreased CD135 staining on cDC2 from *Clec9a^Cre/Cre^Rosa^DTA^* mice may reflect the fact that the receptor is engaged by FLT3L, as well as a possible reduction in gene expression. We next used cDC and macrophage core gene signatures (65) and performed unsupervised hierarchical clustering of cDC2 from *Clec9a^Cre/Cre^Rosa^DTA^* mice and YFP^+^ cDC2 in the context of published datasets for cDC and macrophage populations from the Immgen database. When compared in this manner cDC2 from *Clec9a^Cre/Cre^Rosa^DTA^* mice and YFP^+^ cDC2 showed greater similarity to splenic cDC2 than to cDC1 or red pulp macrophages (Figures 5A,B; Figure S3 in Supplementary Material). cDC2 from *Clec9a^Cre/Cre^Rosa^DTA^* mice did not display a migratory cDC signature (Figure S4 in Supplementary Material), suggesting that they are not cDC2 that have aberrantly migrated from the periphery.

We next performed principal component analysis (PCA) in the context of published datasets for splenic cDCs, thymic cDCs, migratory cDCs, splenic pDCs, macrophages, and monocytes. Notably, cDC2 from *Clec9a^Cre/Cre^Rosa^DTA^* mice were most closely related to YFP^+^ cDC2 and splenic cDC2 and clustered far away from monocytes/macrophages, thymic cDCs, or pDCs (Figure 5C). In humans, a subset of cDC2 has been identified that appears to be related to pDCs (66). These cells can be distinguished from cDC2 and pDCs by expression of signature genes, including Axl, Pp1rb, Dab2, CD22, and IL3Ra (66). However, we found no enrichment of the Axl gene signature in cDC2 from *Clec9a^Cre/Cre^Rosa^DTA^* mice compared with YFP^+^ cDC2 (Figure 5D). Taken together, these data demonstrate that cDC2 from *Clec9a^Cre/Cre^Rosa^DTA^* mice do not show a comprehensive loss of cDC identity and resemble cDC2 from wild type mice.

CLPs can give rise to cDCs *in vivo* and *in vitro* under certain conditions (41–48) and pDCs in normal mice can originate from myeloid or lymphoid progenitors that appear phenotypically indistinguishable except for a history of RAG expression in those originating from the latter (50, 67–69). We therefore sorted CD11b^+^CD11c^+^MHCII^+^ cells from control and *Clec9a^Cre/Cre^Rosa^DTA^* mice (Figures 5E,F; Figures S5A–C in Supplementary Material) and performed PCR analysis for Ig gene rearrangements indicative of historical RAG activity. Sorted pDCs from control mice exhibited prominent Ig gene rearrangements (Figures 5E,F; Figure S5C in Supplementary Material), as reported (49, 50), serving as a positive control. Strikingly, cDC2 from *Clec9a^Cre/Cre^Rosa^DTA^* mice also showed clear evidence of Ig gene rearrangements, which were completely absent in cDC2 from control mice (Figures 5E,F; Figure S5C in Supplementary Material). In summary, cDC2 that arise in the absence of *Clec9a*-expressing cDC progenitors are transcriptionally similar to bona fide cDC2 but they exhibit RAG gene expression history in the form of D–J somatic rearrangements, indicating a putative lymphoid origin.

## Discussion

Although the existence of a lymphoid path to cDC development in mice has been suggested (43, 47, 49, 50, 70), in steady-state cDCs arise primarily from myeloid progenitors (53). Here, we report the generation of *Clec9a^Cre/Cre^Rosa^DTA^* mice as a model to constitutively deplete *Clec9a*-expressing cDC progenitors and their progeny. We show that in the absence of these myeloid-derived cDC progenitors, cells that are phenotypically and transcriptionally similar to bona fide cDC2 develop in spleen. These cells show RAG gene expression history in the form of D-J rearrangements at the IgH locus indicating a relation to the lymphoid branch of hematopoiesis. Therefore, our study suggests that lymphopoiesis can contribute to the generation of cDC-like cells in a situation in which myeloid cDC progenitors are impaired. This observation opens an interesting conundrum, namely, whether such cells should be considered part of the cDC lineage. We and others have proposed that mononuclear phagocytes should predominantly be defined on the basis of their ontogeny (12, 71, 72). However, environmental imprinting appears to be a major identity determining criterion for mononuclear phagocytes (73–77). Therefore, an ontogenetic view to cell definition may be less tenable if cell populations with different origins turn out to be identical by all measures examined. Because cDC2 in *Clec9a^Cre/Cre^Rosa^DTA^* mice are not CDP-derived but show lymphoid gene expression history, we suggest to refer to these cells as lymphoid DC2 for the time being (12). Although we find strong similarities between cDC2 and lymphoid DC2, additional studies are necessary to further define the transcriptional and growth factor requirements, as well as functional properties of lymphoid DC2 to firmly establish their lineage affiliation and determine whether these cells should be considered a new type of mononuclear phagocyte (12).

As cDC differentiation from precursors is thought to be homeostatically regulated by the size of the mature cDC pool (78, 79), we cannot formally exclude at this point that some CDPs escape deletion in *Clec9a^Cre/Cre^Rosa^DTA^* mice to preferentially expand and contribute to the cDC2 population. However, our data demonstrate that at least part of the cDC2 pool in *Clec9a^Cre/Cre^Rosa^DTA^* mice derives from a cell type that has undergone Ig gene rearrangement and that pool is therefore qualitatively distinct from CDP-derived cDC2. Whether these ontogenetically distinct cDC2s arise from early lymphoid-committed progenitors, such as CLPs, is an attractive possibility that will need to be explored. However, it is worth mentioning that some myeloid progenitors reportedly express RAG1 and can generate pDCs with D-J rearrangements *in vitro* (50). Such RAG1-expressing myeloid progenitors, which would presumably lack DNGR-1, could contribute to cDC2 generation in *Clec9a^Cre/Cre^Rosa^DTA^* mice, in which case our designation of these cells as “lymphoid cDC2” would be incorrect. Finally, it is possible that pDCs, some of which have a lymphoid past (49, 50), could convert to lymphoid cDC2 in the FLT3L-rich environment of *Clec9a^Cre/Cre^Rosa^DTA^* mice, although this is difficult to reconcile with the fact that we failed to find any pDC-specific transcripts in lymphoid DC2 (Figure 5 and unpublished observations). Another intriguing observation is that lymphoid DC2 display decreased surface staining for CD135. Gene expression analysis suggests that CD135 may be dysregulated at the RNA level (Figure 5). However, it is equally possible that CD135 could be downregulated upon continuous receptor engagement by FLT3L (80) or may no longer be available for antibody binding (Figure S3D in Supplementary Material). As FLT3L-deficient mice lack all cDCs (33, 81), it is unlikely that lymphoid DC2 arise independently of FLT3L. It will be important to determine whether different growth factors may preferentially promote the differentiation of lymphoid and myeloid progenitors into cDCs.

The fact that lymphoid DC2 are strikingly similar to bona fide cDC2 of myeloid origin in terms of transcriptional profile is in agreement with data demonstrating that *in vitro* generated human myeloid-derived cDC1 are indistinguishable from ones derived from human lymphoid-committed progenitors (42). These data suggest a primary role for the environment in the functional imprinting of cDCs, similar to observations made for macrophages and monocytes (73–76). Despite functional similarities between lymphoid DC2 and cDC2 *in vitro*, these cells respond somewhat differently to the TLR ligand LPS. These data raise the possibility that lymphoid DC2 are not fully functionally equivalent to cDC2, which needs to be addressed experimentally in more detail. In this context, it is noteworthy that *Clec9a^Cre/Cre^Rosa^DTA^* mice develop signs of systemic myeloproliferation, which is typical of mice lacking both cDC1 and cDC2 but has not been reported in mice lacking only the cDC1 subset (82, 83).

It will be important to determine whether additional situations exist where a lymphoid path may contribute to generating or replacing bona fide cDC2, either to ensure functional redundancy or to mediate specific immune functions. Notably, RAG-expressing immune-restricted progenitors contribute to embryonic myelopoiesis in the fetal liver and, although this occurs before the onset of definitive hematopoiesis (84), it indicates that lymphoid and myeloid lineage decisions are not always binary. During emergency hematopoiesis distinct monocyte progenitors have been suggested to respond to inflammatory stimuli on a per need basis, yielding a situation-adapted repertoire of inflammatory monocytes (85). It is intriguing to speculate that, in a similar manner, lymphoid and myeloid progenitors may be differentially triggered in certain conditions of inflammation to generate lymphoid DC2 and cDC2, respectively.

## Ethics Statement

All animal experiments were performed in accordance with national and institutional guidelines for animal care and approved by the Francis Crick Institute Animal Ethics Committee, the UK Home Office, or the Regierung of Oberbayern.

## Author Contributions

JS and BS planned and performed experiments. JB, NP, SR, MP, DP, NR, and SJK performed experiments. TS performed Microarray analysis. JS, BS, and CRS wrote the manuscript. BS and CRS designed the study.

## Conflict of Interest Statement

The authors declare that the research was conducted in the absence of any commercial or financial relationships that could be construed as a potential conflict of interest.
