# Supplementary material for: Clec9a-Mediated Ablation of Conventional Dendritic Cells Suggests a Lymphoid Path to Generating Dendritic Cells In Vivo
Source: Front Immunol. 2018 Apr 16;9:699. doi: 10.3389/fimmu.2018.00699 (PMC5911463; doi:10.3389/fimmu.2018.00699)
Supplement: Supplementary file 1 [file data_sheet_1.PDF]

Supplementary Material

Clec9a-mediated ablation of conventional dendritic cells suggests a lymphoid path to generating dendritic cells *in vivo*

Johanna Salvermoser[1,2], Janneke van Blijswijk[3], Nikos E. Papaioannou[2], Stephan Rambichler[1,2], Maria Pasztoi[2], Dalia Pakalniškytė[1,2], Neil C. Rogers[3], Selina J. Keppler[4], Tobias Straub[2,5], Caetano Reis e Sousa[3*], Barbara U. Schraml[1,2*]

[1] *Walter-Brendel-Centre for Experimental Medicine, University Hospital, LMU Munich, Großhaderner Str. 9, 82152 Planegg Martinsried, Germany*
[2] *Biomedical Center, LMU Munich, Großhaderner Str. 9, 82152 Planegg Martinsried, Germany*
[3] *Immunobiology Laboratory, The Francis Crick Institute, 1 Midland Road, London NW1 1AT, United Kingdom*
[4] *Technische Universität München, Klinikum Rechts der Isar, Institut für Klinische Chemie und Pathobiochemie, Ismaningerstr. 22, 81675 Munich, Germany*
[5] *Core Facility Bioinformatics, Biomedical Center (BMC), LMU Munich, Großhaderner Str. 9 82152 Planegg Martinsried*
**These authors contributed equally*

correspondence: barbara.schraml@med.uni-muenchen.de , caetano@crick.ac.uk

Supplementary Figure 1

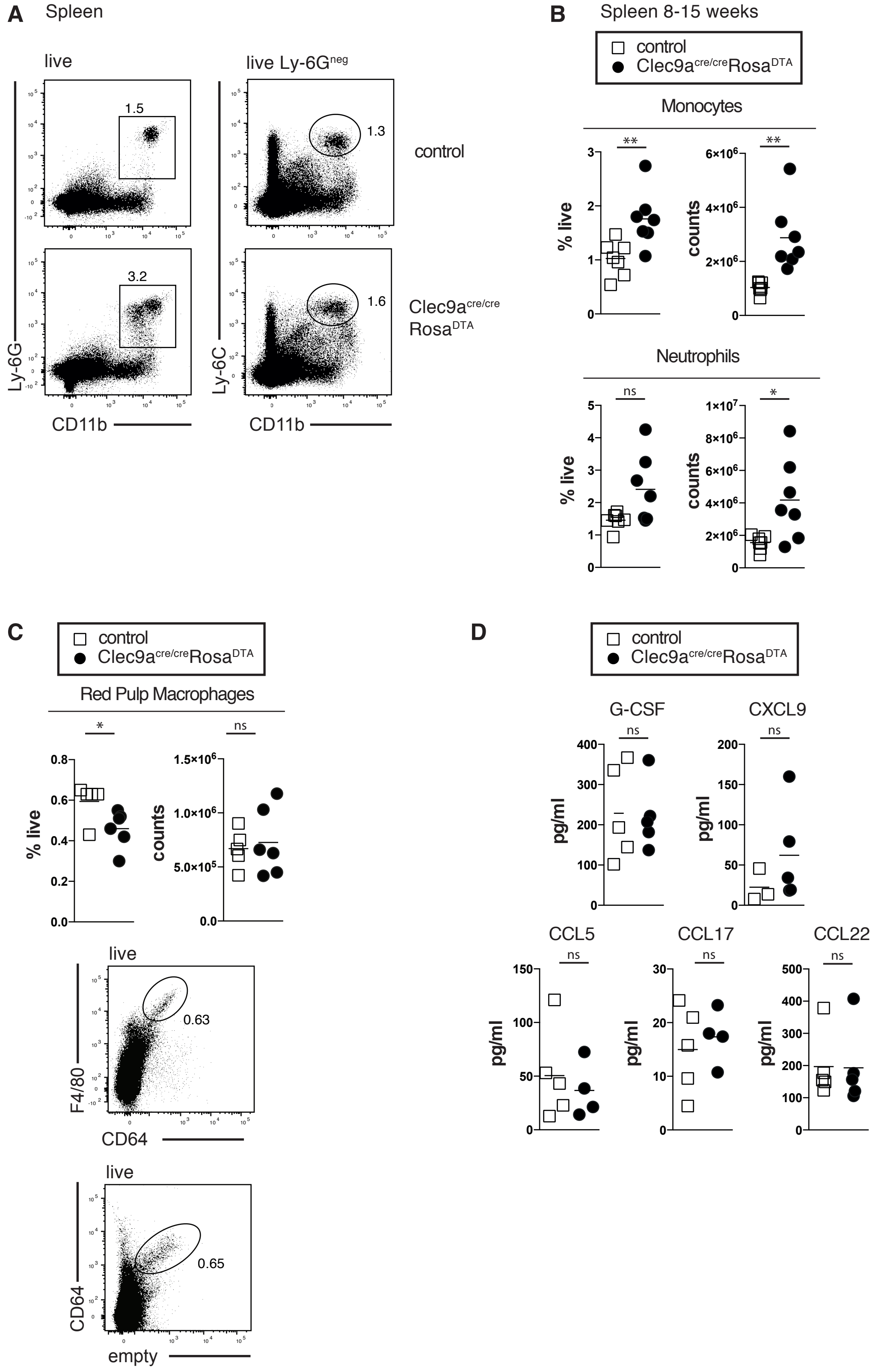

Supplementary Figure 1: *Clec9a$^{cre/cre}$Rosa^{DTA}* mice develop signs of age-dependent myeloproliferation

A, B. Neutrophils and monocytes were identified by flow cytometry in spleen from control and *Clec9a$^{cre/cre}$Rosa^{DTA}* mice. **A**. Representative gating strategy in 14-week-old mice. Neutrophils were identified as live CD11b^{+}Ly-6G^{+} cells. Monocytes were identified as live Ly-6G^{-}Ly-6C^{+}CD11b^{+} cells. **B.** The frequency and total counts of splenic Ly-6G^{+} neutrophils and Ly-6C^{+} monocytes are plotted. **C.** Frequency and counts of red pulp macrophages (RPM) identified as autofluorescent CD64^{+}F4/80^{hi} cells or as autofluorescent CD64^{+} cells in spleen from *Clec9a$^{cre/cre}$Rosa^{DTA}* and control mice. Representative gating strategies to identify red pulp macrophages are shown. **D.** Serum was collected from control and *Clec9a$^{cre/cre}$Rosa^{DTA}* mice older than 10 weeks and the concentrations of G-CSF, CXCL9, CCL5, CCL17, and CCL22 were determined by ELISA or cytometric bead array as described in the materials and methods. Data are compiled from at least two independent experiments. Each symbol represents one mouse. * $p<0.05$, ** $p<0.001$.

Supplementary Figure 2

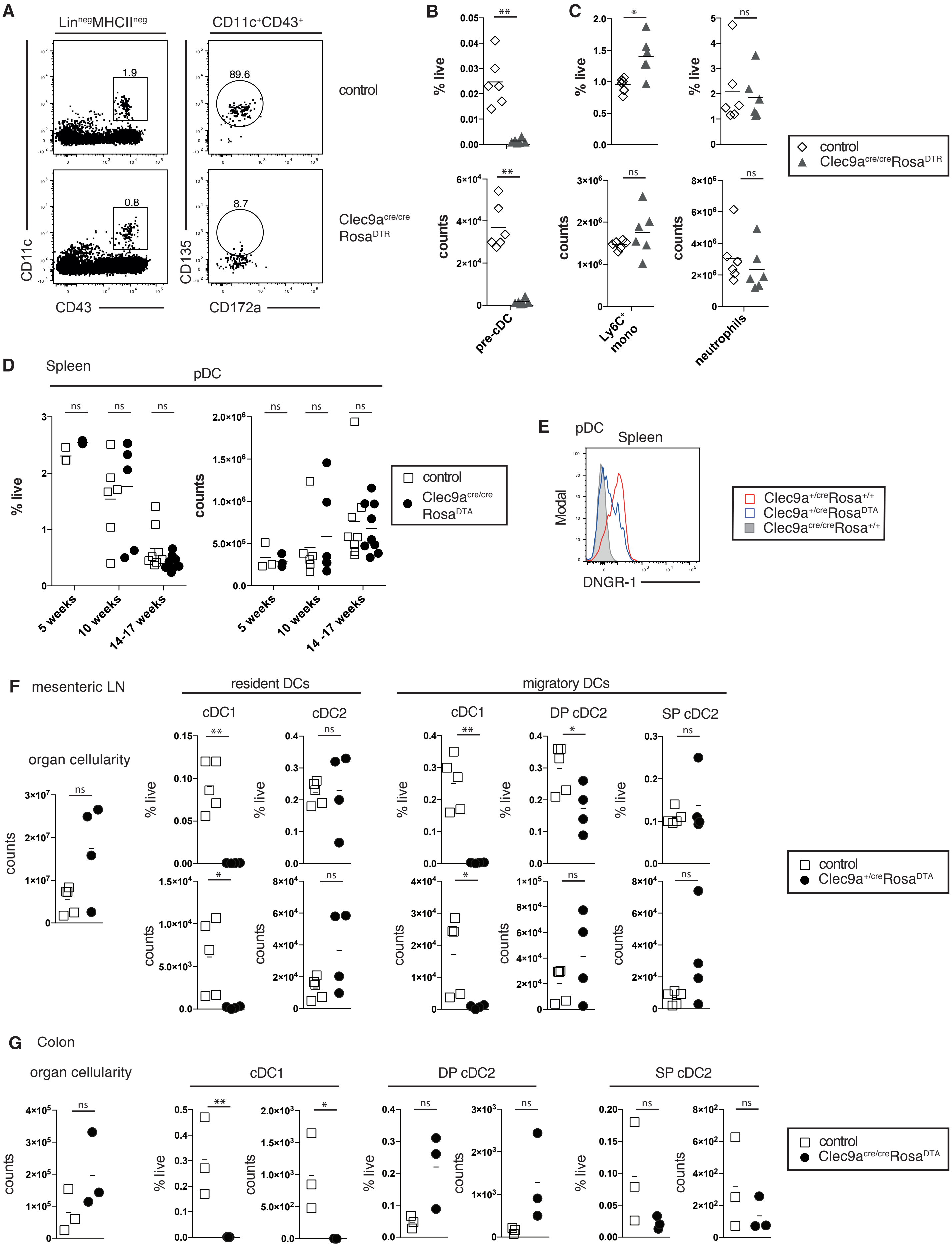

Supplementary Figure 2: Analysis of pDCs in spleen from *Clec9a*$^{cre/cre}$*Rosa*^{DTA} mice and loss of pre-cDCs in *Clec9a*$^{cre/cre}$*Rosa*^{DTR} mice

A, B, C. *Clec9a*$^{cre/cre}$*Rosa*^{DTR} and control mice were injected i.p. with 25 ng/g of diphtheria toxin (DT). 24 hours later spleens were analysed by flow cytometry. **A.** Representative gating strategy of splenic lin$^-$ (CD3, CD4, CD8, Ter119, NK1.1, B220, CD11b, MHCII) CD11c$^+$CD43$^+$CD135$^+$CD172a^{low} pre-cDCs. **B.** Frequency and total counts of pre-cDCs were identified as in **(A)**. **C.** The frequency and total counts of neutrophils (Ly-6G$^+$CD11b$^+$) and Ly-6C$^+$ monocytes (Ly-6G$^-$Ly-6C$^+$CD11b$^+$) are shown. **D.** Splenic pDCs were identified as SiglecH$^+$B220$^+$ cells by flow cytometry in *Clec9a*$^{cre/cre}$*Rosa*^{DTA} and control mice. The frequency and counts of splenic pDCs in *Clec9a*$^{cre/cre}$*Rosa*^{DTA} and control mice of the indicated ages are shown. **E.** Analysis of DNGR-1 protein expression on pDCs from *Clec9a*$^{+/cre}$*Rosa*^{DTA} mice. DNGR-1 protein levels were analysed by flow cytometry on splenic B220$^+$SiglecH$^+$ pDCs from *Clec9a*$^{+/cre}$*Rosa*$^{+/+}$ control mice and compared to levels on pDCs from *Clec9a*$^{+/cre}$*Rosa*^{DTA} mice. Homozygous Clec9a$^{cre/cre}$*Rosa*$^{+/+}$ mice serve as a negative control for DNGR-1 protein staining. **F, G.** mesenteric LN from *Clec9a*$^{+/cre}$*Rosa*^{DTA} and control mice **(F)** and colon from *Clec9a*$^{cre/cre}$*Rosa*^{DTA} and control mice **(G)** were analysed for total organ cellularity, as well as for the presence of the indicated DC subsets. **F.** Resident and migratory cDCs were distinguished as CD11c$^+$MHCII$^+$ and CD11c^{int}MHCII^{hi} cells respectively. cDC1 were identified as XCR-1$^+$CD11b$^-$ cells and cDC2 were identified as XCR-1$^-$CD11b$^+$ or CD103$^+$CD11b$^+$ (DP) and CD103$^-$CD11b$^+$ (SP) cDC2. **F.** Colonic cDCs were identified as CD11c$^+$MHCII$^+$CD64$^-$ cells and distinguished as XCR-1$^+$CD172a$^-$ cDC1, as well as CD103$^+$CD11b$^+$CD172a$^+$ (DP) and CD103$^-$CD11b$^+$CD172a$^+$ (SP) cDC2. Each symbol represents one mouse. ns - not significant, *$p<0.05$, ** $p<0.001$.

Supplementary Figure 3

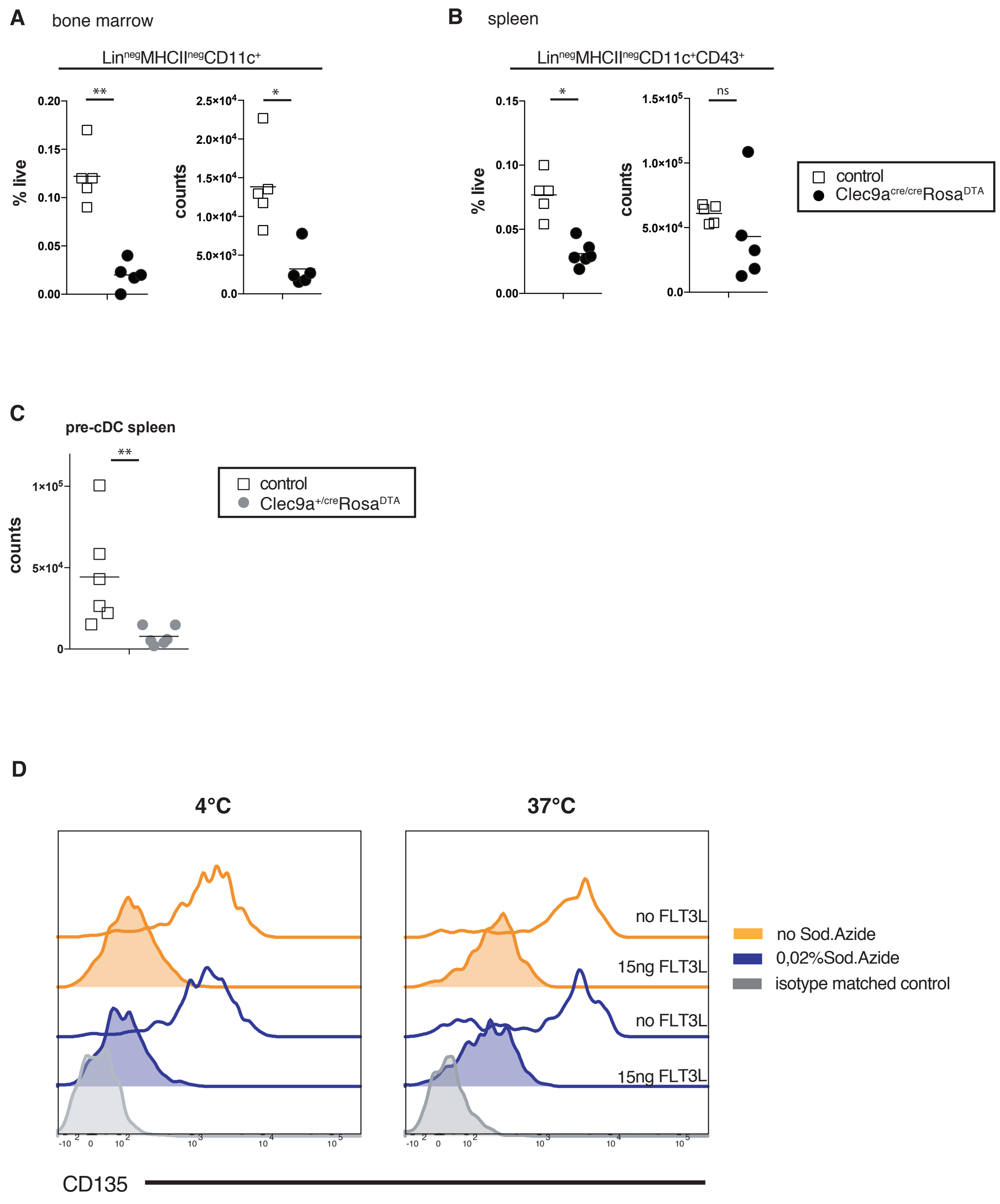

Supplementary Figure 3. Loss of cDC progenitors in *Clec9a*$^{cre/cre}$*Rosa*^{DTA} and *Clec9a*$^{+/cre}$*Rosa*^{DTA} mice.

A, B. cDC progenitors were identified by flow cytometry in the bone marrow and spleen of 10-week-old control and *Clec9a*$^{cre/cre}$*Rosa*^{DTA} mice. **A.** Live lineage negative (lin$^-$; CD3, CD4, CD8, CD11b, MHCII, Ter119, NK1.1, B220) CD11c$^+$ cells from bone marrow of *Clec9a*$^{cre/cre}$*Rosa*^{DTA} and control mice were gated and quantified. The frequency and total counts per femur are shown. **B.** Lin$^-$CD11c$^+$CD43$^+$ splenocytes, containing pre-cDCs, were identified in control and *Clec9a*$^{cre/cre}$*Rosa*^{DTA} mice 10 weeks of age. Frequency and total counts per spleen are shown. **C.** Lin$^-$CD11c$^+$CD43$^+$CD135$^+$CD172a^{low} pre-cDCs were quantified in spleen from *Clec9a*$^{+/cre}$*Rosa*^{DTA} mice 9-12 weeks of age. **D**. CD11c$^+$ splenocytes were cultured with or without 15ng/ml FLT3L for 2 hours at 37°C or 4°C in the presence or absence of 0,02% sodium azide to interfere with endocytosis. Cells were then washed and analysed for CD135 expression. CD135 staining on CD11c$^+$MHCII$^+$CD11b$^+$ splenocytes is shown.

Supplementary Figure 4

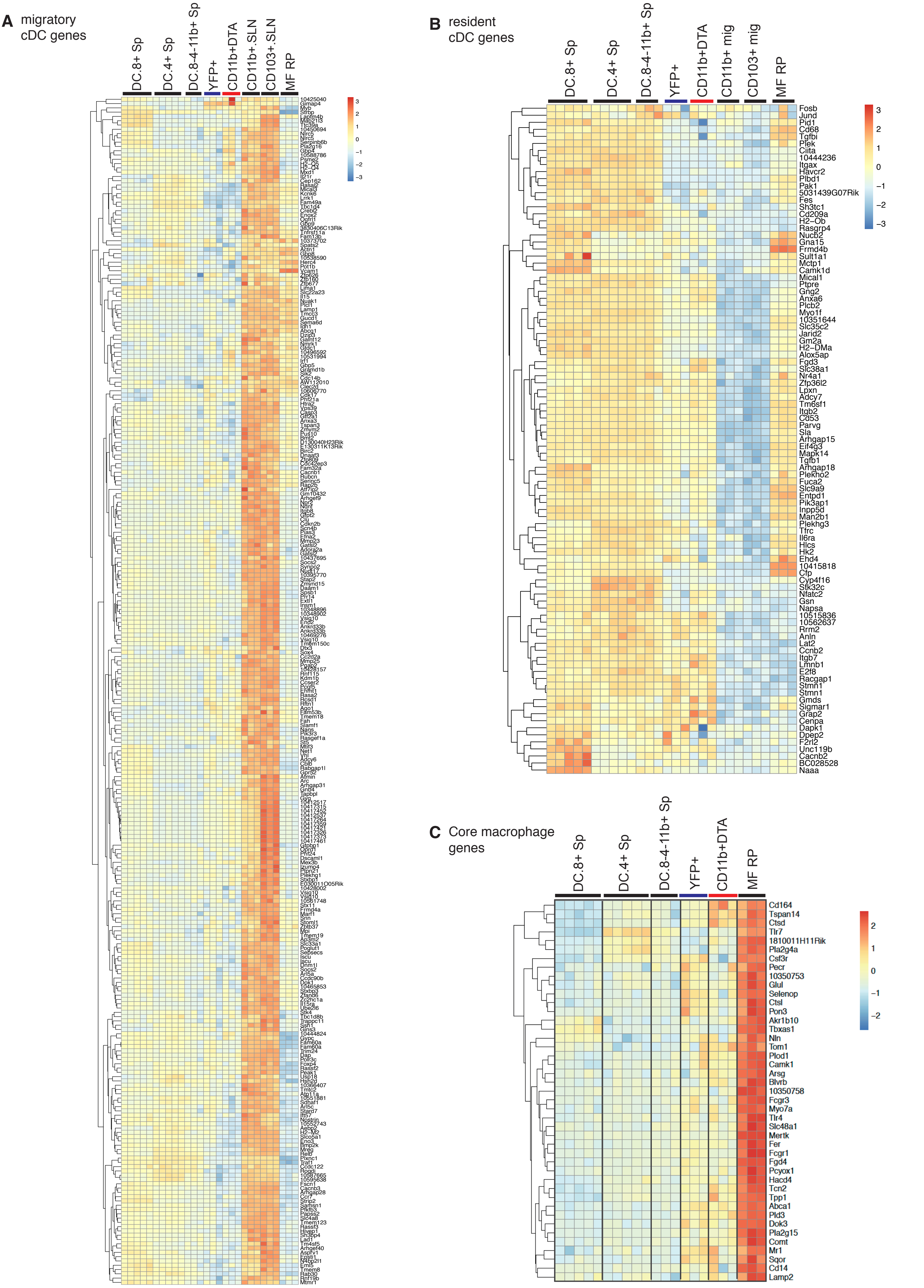

Supplementary Figure 4: Transcriptional profiling of cDC2 from *Clec9a*$^{cre/cre}$*Rosa*^{DTA} mice reveals no major loss in cDC identity.

Splenic CD11c^{+}MHCII^{+}CD11b^{+} cDC2 *Clec9a*$^{cre/cre}$*Rosa*^{DTA} mice (CD11b^{+} *DTA*) and cDC2 (identified as CD11c^{+}MHCII^{+}CD11b^{+}YFP^{+} cells from *Clec9a*$^{cre/cre}$*Rosa*^{YFP} mice; YFP^{+}) were sorted and subjected to microarray analysis. Population clustering and heat map display of the relative expression values for migratory cDC signature genes **(A)**, resident cDC signature genes **(B),** and core macrophage signature genes **(C)** in CD11b^{+} DTA and YFP^{+} cDC2 samples compared to the indicated murine cDC and macrophage populations from the Immgen database.

Supplementary Figure 5

A Sort strategy for cDC2

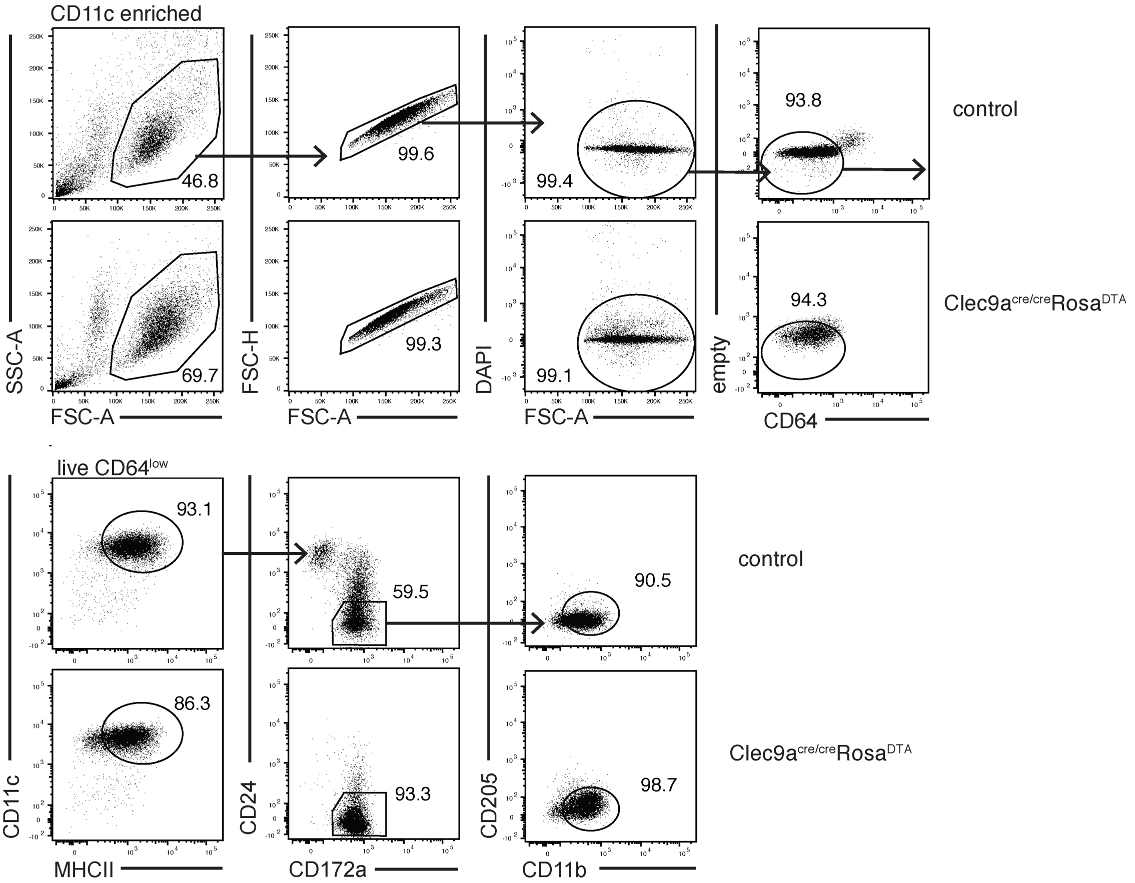

B

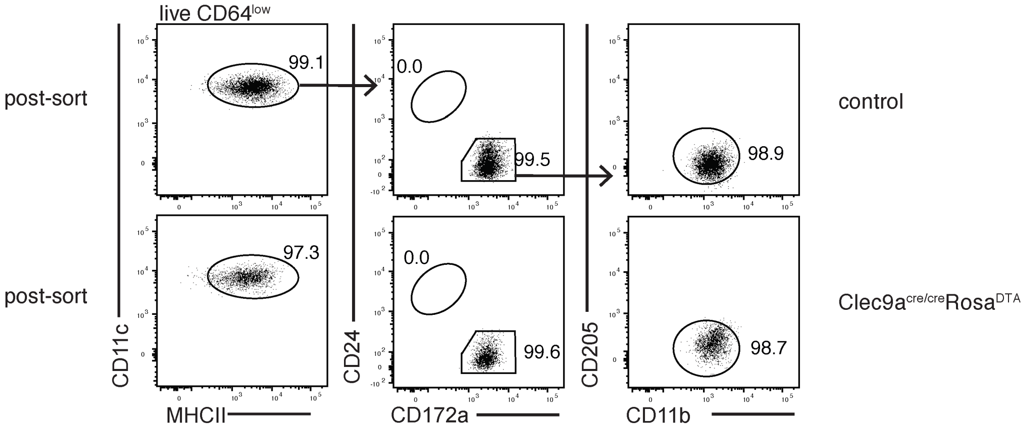

C

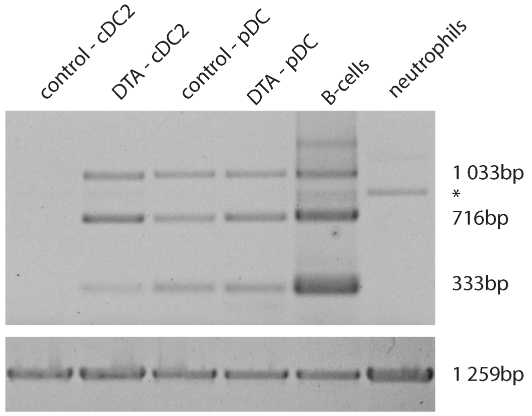

Supplementary Figure 5: cDC2 from Clec9a$^{cre/cre}$Rosa^{DTA} mice exhibit somatic rearrangements of lymphoid receptor genes.

A, B. Gating strategy and purity check for DJ- rearrangement PCR. **A.** CD11c^{+}MHCII^{+}CD11b^{+} cDC2 from control and *Clec9a$^{cre/cre}$Rosa^{DTA}* mice were sorted according to the indicated gating strategy. **B**. The purity of the sorted cDC2 populations from control and *Clec9a$^{cre/cre}$Rosa^{DTA}* mice is shown. **C.** CD11c^{+}MHCII^{+}CD11b^{+} cDC2 from control and *Clec9a$^{cre/cre}$Rosa^{DTA}* mice were sorted and genomic DNA was isolated. Genomic PCR was performed using primers for the germline (GL) locus and primer mixtures homologous for regions of the Dfl16 and Dsp2 D gene families for detecting D–J rearrangements of the IgH chain. DJ rearrangements in pDCs (SiglecH^{+}B220^{+}), splenic B-cells and neutrophils (Ly-6G^{+}) are shown as control. * indicates an unspecific band.
