# Supplementary material for: Clec9a-Mediated Ablation of Conventional Dendritic Cells Suggests a Lymphoid Path to Generating Dendritic Cells In Vivo
Source: Front Immunol. 2018 Apr 16;9:699. doi: 10.3389/fimmu.2018.00699 (PMC5911463; doi:10.3389/fimmu.2018.00699)
Supplement: Supplementary file 3 [file table_2.docx]

Supplementary Table 2: Antibodies used in the study

| Antigen | clone | conjugate | company |
| --- | --- | --- | --- |
| CD3 | 145-2C11 | Pacific Blue (PB), Fluorescein isothiocyanate (FITC) | Biolegend |
| CD3 | 145-2C11 | e-450 | eBioscience |
| CD4 | RMA4.5 | PB | BD-Bioscience |
| CD4 | RM4-5 | PB | Biolegend |
| CD4 | GK1.5 | FITC | Biolegend |
| CD4 | RM4-5 | eF780 | eBioscience |
| CD8 | 53-6.8 | FITC, PB | BD-Bioscience |
| CD8 | 53-6.8 | FITC, PB | Biolegend |
| CD8 | 53-6.8 | eFluor605nc | eBioscience |
| CD11b | M1/70 | FITC, APC | BD-Bioscience |
| CD11b | M1/70 | FITC, PB, APC Cy7 | Biolegend |
| CD11b | M1/70 | AF700, APC eFluor780, PB | eBioscience |
| CD11c | N418 | PerCp (Peridinin-chlorophyll-protein) Cy5.5, APC/Cy7 | Biolegend |
| CD16/32 | 2.4G2 | Purified (Fc-Block) | BD-Bioscience |
| CD16/32 | 93 | FITC | Biolegend |
| CD19 | ID3 | PE | BD-Bioscience |
| CD24 | M1/69 | BV605 | BD-Bioscience |
| CD24 | M1/69 | BV605 | Biolegend |
| CD43 | 1B11 | PeCy7, APC | Biolegend |
| B220 (CD45R) | RA3-6B2 | FITC, PE, PB | BD-Bioscience |
| B220 (CD45R) | RA3-6B2 | FITC, PE, PB | Biolegend |
| CD64 | X54-5/7.1 | PE, PeCy7 | Biolegend |
| CD45.2 | 104 | AF700 | eBioscience |
| CD115 | AFS98 | BV605, APC | Biolegend |
| CD115 | AFS98 | AF488, APC | eBioscience |
| CD117 | 2B8 | PerCpCy5.5, PeCy7 | Biolegend |
| CD135 | A2F10 | PE, APC | Biolegend |
| CD135 | A2F10 | PerCPeF710 | eBioscience |
| NK1.1 (CD161c) | PK136 | PB, FITC | Biolegend |
| CD172a | P84 | PE, APC | BD-Bioscience |
| CD172a | P84 | PeCy7 | Biolegend |
| CD205 | NLDC-145 | APC | Biolegend |
| MHCII I-A/I-E | M5/114.15.2 | FITC, AF700, PB | Biolegend |
| MHCII I-A/I-E | M5/114.15.2 | E450, APC eFluor780, AF700, FITC | eBioscience |
| MHCII I-A/I-E | M5/114.15.2 | E450 | BD-Bioscience |
| SiglecH | eBio440c | eF660 | BD-Bioscience |
| SiglecH | eBio440c | AF647, eF660 | eBioscience |
| SiglecH | 551 | PerCpCy5.5 | Biolegend |
| F4/80 | C1:A3-1 | AF647, BV785 | Biolegend |
| ESAM |  |  |  |
| Clec4a4 | 33D1 | APC | Biolegend |
| DNGR-1 | 1F6 | Custom conjugated with PE | eBioscience |
| Ter119 | Ter-119 | FITC, PB | Biolegend |
| Ly6G | RB68C5 | AF700 | eBioscience |
| Ly6G | 1A8 | PB, AF700 | Biolegend |
| Ly6C | HK1.4 | BV605, PB | Biolegend |
| TNFα | MP6-XT22 | PeCy7 | eBioscience |
| IL-12/IL23 p40 | C17.8 | PE | eBioscience |
| IRF8 | V3GYWCH | PE | eBioscience |
| IRF4 | M17 |  | Santa Cruz Biotechnology |
| Zbtb46 | U4-1374 | PE | BD-Bioscience |
| Donkey Anti-goat IgG |  | AF647 | ThermoFisher |
| Isotype (mIgG1, κ) | MOPC-21 | PE | Biolegend |
| Isotype (rIgG1, κ) | R3-34 | PE | BD-Bioscience |
| Isotype (rIgG2a, κ) | RTK2758 | PE | Biolegend |
